# Reactogenicity of COVID-19 Vaccines in Patients With a History of COVID-19 Infection: A Survey Conducted in Pakistan

**DOI:** 10.7759/cureus.31359

**Published:** 2022-11-11

**Authors:** Muhammad Riyyan, Hafiz A Sarwar, Hassan A Chania, Sawaira Sajid, Sonika Hotwani, Hafiz U Sarwar, Sheeza Nawaz, Shariq Abid

**Affiliations:** 1 Medical Research Center, Liaquat University of Medical and Health Sciences, Jamshoro, PAK; 2 Medicine and Surgery, Islam Medical College, Sialkot, PAK

**Keywords:** vaccine side effects, viral vector vaccine, inactivated virus vaccine, sars-cov-2 vaccine reactogenicity, covid-19 infection, mrna-based vaccine

## Abstract

Introduction

As coronavirus disease 2019 (COVID-19) immunizations become more common, concerns about their safety and reactogenicity have grown. It is important to assess and analyze the post-vaccination side effects of several COVID-19 vaccines that have been licensed in Pakistan.

Methods and results

A comparative cross-sectional study was conducted between October 2021 and January 2022 to collect data on the side effects produced by different COVID-19 vaccines. An online survey was conducted to gather data on participants' demographics, clinical profiles, COVID-19 profiles as well as the intensity and side effects of COVID-19 vaccines. Statistical Package for the Social Sciences (SPSS) version 22.0 (IBM Corp., Armonk, NY) was used to analyze the data collected. Out of 421 participants, 63.2% were males, 36.8% of participants received messenger RNA (mRNA) vaccine, 33.2% received viral vector vaccine, 29.9% received inactivated vaccine, and further 71.7% of the total subjects were completely immunized. The majority of the symptoms were mild to moderate in degree. Approximately, 0.7% of the individuals reported experiencing serious adverse effects. Injection site pain (35.9%) was noted to be the most remarkable post-vaccination side effect followed by fever (33.2%) and fatigue (23.1%). Prior COVID-19 infection was not associated with the severity of any COVID-19 vaccine-related side effect (p* *> 0.05), except dyspnea. Younger participants and the female gender were substantially linked to post-vaccination adverse effects.

Conclusion

In comparison to viral vector and inactivated vaccines, our data suggest that the mRNA-based vaccination causes more severe adverse effects, and the majority of them were mild to moderate in severity. Participants who had previously contracted COVID-19 were not at a higher risk of developing additional vaccine-related side effects.

## Introduction

The coronavirus disease 2019 (COVID-19), or severe acute respiratory syndrome coronavirus 2 (SARS-CoV-2), has infected tens of millions of people around the world, posing a serious threat to public health as well as social and economic well-being [1]. With a total of 1,502,641 confirmed cases and nearly 30,053 deaths to date in Pakistan [2], this disease has been one of the biggest challenges. Safe and effective vaccinations appear to be a more efficacious way to contain the pandemic than other therapeutical methods that have been tested as a major decline in COVID-19 cases was observed among the Israeli population during the peak of the COVID-19 pandemic by initiating a rapid vaccination drive, which also helped in minimizing transmission [3]. Pakistan started its vaccination program in February 2021, starting with frontline healthcare workers who were at a high risk of contracting COVID-19. Currently available vaccines against SARS-CoV-2 in Pakistan are based on one of the following technologies: (a) mRNA-based vaccines, (b) viral vector-based vaccines, and (c) whole virus or inactivated virus vaccines [4]. In medical literature, vaccine hesitancy is defined as “refusal to get vaccinated or delayed acceptance even when vaccines are available” [5]. One of the frequent causes of this hesitancy is skepticism regarding the potential side effects of vaccines [6]. Lack of awareness regarding vaccine reliability and adverse effects leads to hesitancy toward vaccines [7]. A study conducted in the United Kingdom suggested that fear regarding adverse events related to vaccines is the most remarkable reason for vaccine hesitancy [8].

Pakistan has faced multiple waves of COVID-19 due to an upsurge of various types of COVID-19 variants. With the ongoing fifth wave, omicron and delta are the variants being detected [9]. Although vaccines appear to be less effective against the delta variant than the alpha strain, they are still highly effective in preventing disease after two doses [10]. A study conducted in South Africa showed that the COVID-19 vaccine produced by Pfizer is still efficacious against the omicron variant [11]. The omicron variant bypasses the natural immunity induced by COVID-19 infection and is linked to a higher risk of reinfection [12]. In the United States, the first death related to the omicron variant was of a non-vaccinated individual with a history of COVID-19 [13].

The primary goal of this study is to analyze the reactogenicity and safety profiles of different types of vaccines available in Pakistan. Moreover, another aim of this study is to investigate whether there is a link between the severity and frequency of vaccine-related side effects and previous COVID-19 infection.

## Materials and methods

Study design, period, and area

A comparative cross-sectional study was conducted in Pakistan from October 2021 to January 2022. The study was designed for individuals of every age above 12 years and living in any province of the county. An online survey designed on Google forms was used for data collection through social media platforms. The data collection was done in December 2021. A total of 472 participants who were Pakistani citizens from every province of the country responded, out of which 421 participants who met the inclusion criteria were included in this study.

Inclusion and exclusion criteria

The inclusion criteria of the study included Pakistani citizens aged ≥12 years and those who had been vaccinated with either single or double doses of the Pakistani authorities-approved COVID-19 vaccines. Exclusion criteria included participants who were diagnosed with COVID-19 infection with positive antibody tests. Participants who submitted incorrect/incomplete forms were also excluded from the study.

Sample size determination and sampling procedure

Purposive sampling was used as a sampling method for this study. Data were collected from all over Pakistan via social media platforms and professional contacts. A sample size of 421 was calculated using the OpenEpi sample size calculator and keeping a level of significance 95% confidence interval, and 3.5% error, and a study conducted by the Centers for Disease Control and Prevention (CDC) was used to determine the predicted prevalence of fever, which was found to be 15.8% [14].

Study variables and measurements

A standardized self-administered online questionnaire (SAQ) created on Google forms was utilized to collect the data concerning this study. The questionnaire included four major sections in sequence: demographic data including age, gender, and residency; clinical profile including comorbidities and smoking history; COVID-19 profile including vaccination status, type of vaccine, previous history of infection, diagnostic test, and severity of COVID-19 infection; and severity of side effect of COVID-19 vaccines. The severity scale of COVID-19 infection was developed according to the FDA guidelines.

Data analysis

Data analysis was done by using Statistical Package for the Social Sciences (SPSS) software version 22.0 (IBM Corp., Armonk, NY). Descriptive statistics were carried out for categorical variables like demographic variables (gender, age, and residence), health status (smoking status and comorbidities), COVID-19-related anamnesis (history of COVID-19 infection, severity of infection, and diagnostic test for infection), and COVID-19 vaccination status (number of doses and types of vaccine), which were represented by frequencies (n) and percentages (%). Continuous variable like the age of participants is represented by mean (μ). Consequently, a chi-square test (χ^2^) was performed to assess the statistical significance between (1) different types of COVID-19 vaccines and the severity of self-reported side effects as well as (2) the prior history of COVID-19 infection and the severity of post-vaccination side effects. Binary logistic regression was used to evaluate the incidence of side effects of COVID-19 vaccines among the participants. All inferential tests were carried out assuming a confidence interval (CI) of 95% and a significance value of <0.05.

Ethical approval

This study was carried out after approval from the Research Ethics Committee of Liaquat University of Medical and Health Sciences (LUMHS/REC/-159). Data were collected from the participants who willingly gave consent to participate in this study.

## Results

Between September and November 2021, 471 participants completed the questionnaire; however, only 421 matched the criterion for inclusion. As illustrated in Table 1, the study was carried out on a nationwide scale, with the majority of participants from Sindh (44.9%). The participants' age ranged from 13 to 85 years old, with a median of 33 years old, and 51.8% of participants were ≤33 years. This median age was used to bifurcate the participants into two age groups/variables. Age groups were used to stratify the participants inoculated with different types of COVID-19 vaccines into young and old individuals. Males made up a major share of the participants (63.2%). With regard to health status, most respondents reported no comorbidities (68.6%) and were nonsmokers (87.6%). By the time of filling out the online questionnaire, 71.9% of participants had been fully vaccinated, which includes 120 each for inactivated and mRNA vaccines while only 62 participants were of the viral vector. The majority of the participants who received a single dose of the COVID-19 vaccine were vaccinated with the viral vector-based vaccine.

**Table 1:** Demographic characteristics of the study population * denotes Khyber Pakhtunkhwa, and ** denotes reverse transcriptase polymerase chain reaction test.

Variables		Frequency	Percentage
Gender	Male	266	63.2
	Female	155	36.8
Age groups	≤33 years old	218	51.8
	>33 years old	203	48.2
Residence	Sindh	189	44.9
	Punjab	60	14.3
	KP	52	12.4
	Islamabad	41	9.7
	Baluchistan	45	10.7
	Gilgit-Baltistan	34	8.1
Smoking	Yes	52	12.4
	No	369	87.6
Comorbidities	No comorbidities	289	68.6
	Comorbidities	132	31.4
History of COVID-19 infection	No	318	75.5
	Yes	103	24.5
Vaccination status	Type of vaccine		
Single dose	mRNA	35	29.4
	Inactivated	6	5.0
	Viral vector	78	65.5
Double dose	mRNA	120	39.7
	Inactivated	120	39.7
	Viral vector	62	20.5
Diagnostic test for COVID-19 infection	RT-PCR**	103	21.9
	No history	317	67.3
	Antibody	51	10.8
Severity of COVID-19 infection	Asymptomatic	21	20.4
	Mild	48	46.6
	Moderate	21	20.4
	Severe	7	6.8
	Critical	6	5.8

Demographic characteristics including vaccination status, history of COVID-19 infection, age groups (formed according to the median age), and gender of subjects were stratified according to the type of COVID-19 vaccines inoculated in the subjects as shown in Table 2. A greater number of participants who received mRNA-based vaccines belonged to the age group ≤33 years. Out of the participants who received inactivated COVID-19 vaccine, majority of them were males and were >33 years old. Recipients who received viral vector-based vaccines majorly were male.

**Table 2:** Demographic characteristics stratified by the type of vaccine

Variables		Type of vaccine			Total	Significance
		Inactivated	mRNA	Viral vector		
Vaccination status of subjects	Single dose	6 (4.8%)	35 (22.6%)	78 (55.7%)	119 (28.3%)	0.00%
	Double dose	120 (95.2%)	120 (77.4%)	62 (44.3%)	302 (71.7%)	
History of COVID-19 infection	Yes	70 (55.6%)	20 (12.9%)	13 (9.3%)	103 (24.5%)	0.00%
	No	56 (44.4%)	135 (87.1%)	127 (90.7%)	318 (75.5%)	
Age groups of subjects (years)	≤33	57 (45.2%)	92 (59.4%)	69 (49.3%)	218 (51.8%)	0.05%
	>33	69 (54.8%)	63 (40.6%)	71 (50.7%)	203 (48.2%)	
Gender of subject	Male	77 (61.1%)	85 (54.8%)	104 (74.3%)	266 (63.2%)	0.02%
	Female	49 (38.9%)	70 (45.2%)	36 (25.7%)	155 (36.8%)	

Regarding vaccines type, 88 participants (20.9%) received Pfizer-BioNTech (BNT162b2) vaccine, and 67 participants (15.9%) received Moderna (CX-024414) vaccine (mRNA-based, vaccine n = 155). Twenty-six participants (6.1%) received CanSino Bio (Ad 5-nCoV), 63 (15.0%) received Sputnik V, 49 (11.6%) received Oxford-AstraZeneca (AZD1222), and two participants (0.5%) received PakVac (Ad 5-nCoV) vaccine (viral vector-based vaccine, n = 140). Seventy-one participants (16.9%) received Sinopharm (BBIBP-CorV), and 55 (13.1%) received SinoVac (CoronaVac) vaccine (inactivated-based vaccine, n = 126).

Out of 421 participants included in our study, 103 (24.5%) reported a history of COVID-19 infection; the majority of them were the recipients of inactivated vaccines. According to the severity scale explained in Table 3, 21 participants had an asymptomatic infection, 48 had a mild disease that made up the majority, 21 had moderate clinical outcome, seven reported severe infection and required hospitalization with supplementary oxygen, and only six progressed to a critical stage and required ventilator support.

**Table 3:** Definitions of the severity of side effects

Severity	Definition
Mild	No treatment needed
Moderate	Needed treatment or advice from healthcare professional outside the hospital
Severe	Needed hospital care

According to the observed results, the most remarkable post-vaccination side effect was injection site pain (N = 151 [35.9%]) followed by fever (N = 140 [33.2%]). Fatigue (N = 97 [23.1%]) ranked third, followed by muscle pain (N = 51 [12.1%]), headache (N = 47 [11.2%]), injection site swelling (N = 44 [10.5%]), joint pain (N = 26 [6.2%]), flu-like illness (N = 22 [5.2%]), chills (N = 14 [3.3%]), hypotension (N = 13 [3.1%]), menstrual cycle disturbance (N = 12 [2.8%]), nausea and vomiting (N = 11 [2.6%]), cough (N = 10 [2.4%]), allergic reaction (N = 9 [2.1%]), breathlessness (N = 7 [1.6%]), tachycardia (N = 7 [1.6%]), and diarrhea (N = 5 [1.2%]).

The prevalence of self-reported post-vaccination adverse effects was calculated according to the gender and age of the participants, and the results are shown in Tables 4, 5. Females were more likely to experience the majority of the vaccine-related side effects compared to males like chills (p = 0.03), tachycardia (p = 0.007), hypotension (p = 0.002), fatigue (p = 0.003), muscle pain (p = 0.011), joint pain (p = 0.023), breathlessness (p = 0.007), diarrhea (p = 0.003), nausea and vomiting (p = 0.002); headache (p < 0.001), injection site swelling (p < 0.001), and injection site pain (p < 0.001), whereas some females (n = 11 [7.1%]) reported mensural cycle disturbance for which no comparison can be done on gender grounds. The participant median age was 33 years old, which was utilized as a cut-off for age-dependent comparisons in the analysis of self-reported side effects according to the age of the participants. Younger participants (≤33 years old) were more likely affected by certain side effects compared to older participants (>33 years old), which included chills, headaches, injection site pain, and injection site swelling; these associations were statistically significant with p-values of 0.010, 0.039, <0.001, and 0.003, respectively, but the association of age with self-reported side effects was not statistically significant in other remaining side effects.

**Table 4:** COVID-19 vaccine-related side effects stratified by age groups

Side effect	Age groups of subjects (years)		Significance
	≤33	>33	
Fever	79 (36.2%)	61 (30%)	0.178
Chills	12 (5.5%)	2 (1.0%)	0.010
Cough	6 (2.8%)	4 (2.0%)	0.599
Headache	31 (14.2%)	16 (7.9%)	0.039
Flu-like illness	12 (5.5%)	10 (4.9%)	0.790
Hypotension	8 (3.7%)	5 (2.5%)	0.475
				Fatigue	57 (26.1%)	40 (19.7%)	0.117
Muscle pain	32 (14.7%)	19 (9.4%)	0.095
Joint pain	15 (6.9%)	11 (5.4%)	0.533
Breathlessness	5 (2.3%)	2 (1.0%)	0.294
Nausea and vomiting	6 (2.8%)	5 (2.5%)	0.853
Diarrhea	3 (1.4%)	2 (1.0%)	0.711
Injection site pain	98 (45%)	53 (26.1%)	0.000
Injection site swelling	32 (14.7%)	12 (5.9%)	0.003
Allergic reactions	5 (2.3%)	4 (2.0%)	0.819
Tachycardia	4 (1.8%)	3 (1.5%)	0.775
Menstrual cycle disturbances	7 (3.2%)	5 (2.5%)	0.645

**Table 5:** COVID-19 vaccine-related side effects stratified by gender

Side effect	Gender of subject		Significance
	Male	Female	
Fever	81 (30.5%)	59 (38.1%)	0.110
Chills	5 (1.9%)	9 (5.8%)	0.030
Cough	4 (1.5%)	6 (3.9%)	0.124
Headache	18 (6.8%)	29 (18.7%)	0.000
Flu-like illness	10 (3.8%)	12 (7.7%)	0.077
Hypotension	3 (1.1%)	10 (6.5%)	0.002
Fatigue	49 (18.4%)	48 (31%)	0.003
Muscle pain	24 (9.0%)	27 (17.4%)	0.011
Joint pain	11 (4.1%)	15 (9.7%)	0.023
Breathlessness	1 (0.4%)	6 (3.9%)	0.007
Nausea and vomiting	2 (0.8%)	9 (5.8%)	0.002
Diarrhea	0 (0.0%)	5 (3.2%)	0.003
Injection site pain	79 (29.7%)	72 (46.5%)	0.001
Injection site swelling	16 (6.0%)	28 (18.1%)	0.000
Allergic reactions	4 (1.5%)	5 (3.2%)	0.239
Tachycardia	1 (0.4%)	6 (3.9%)	0.007
Menstrual cycle disturbances	0 (0.0%)	12 (7.7%)	0.000

The severity and incidence of self-reported vaccine side effects were compared between participants with and without a history of COVID-19 infection as summarized in Tables 6, 7. A prior COVID-19 infection was significantly associated with an increased risk of experiencing hypotension (RR 3.602, 95% CI [1.239-10.475]). Although statistically nonsignificant, recipients having a history of COVID-19 infection were shown to have a higher incidence of self-reported cough, headache, flu-like illness, breathlessness, nausea and vomiting, diarrhea, allergic reactions, tachycardia, and menstrual cycle disturbances. The chi-square test revealed that prior COVID-19 infection was statistically not associated with the severity of any COVID-19 vaccine-related side effect (p-value > 0.05), except breathlessness (p = 0.046).

**Table 6:** Incidence of self-reported side effects after COVID-19 vaccination among participants who had or did not have a history of COVID-19 infection

Side effect	Incidence of side effects: risk ratio (RR)	95% Confidence level
Fever	0.952	0.691-1.312
Cough	2.058	0.592-7.152
Headache	1.059	0.571-1.962
Flu-like illness	1.158	0.465-2.881
Chills	0.842	0.240-1.046
Hypotension	3.602	1.239-10.475
Fatigue	0.853	0.555-1.310
Muscle pain	0.662	0.334-1.132
Joint pain	0.561	0.198-1.591
Breathlessness	4.117	0.937-18.09
Nausea and vomiting	1.764	0.527-5.98
Diarrhea	4.61	0785-27.334
Injection site pain	0.83	0.602-1.144
Injection site swelling	0.794	0.395-1.596
Allergic reactions	1.544	0.393-6.063
Tachycardia	4.117	0.937-18.09
Menstrual cycle disturbances	2.205	0.715-6.799

**Table 7:** Difference between the severity of self-reported side effects after COVID-19 vaccination among participants who had or did not have a history of COVID-19 infection

Side effect	History of COVID-19 infection								P-values
	No history of COVID-19 infection				History of COVID-19 infection				
	No side effect	Mild	Moderate	Severe	No side effect	Mild	Moderate	Severe	
Cough	312 (98.1%)	4 (1.3%)	2 (0.6%)	0 (0%)	99 (96.1%)	4 (3.9%)	0 (0%)	0 (0%)	0.174
Fever	211 (66.4%)	47 (14.8%)	60 (18.9%)	0 (0%)	70 (68%)	17 (16.5%)	16 (15.5%)	0 (0%)	0.718
Chills	308 (96.9%)	8 (2.5%)	2 (0.6%)	0(0%)	99 (96.1%)	3 (2.9%)	1 (1%)	0 (0%)	0.914
Flu-like illness	303 (95.3%)	8 (2.5%)	7 (2.2%)	0 (0%)	96 (93.2)	5 (4.9%)	2 (1.9%)	0 (0%)	0.487
Fatigue	242 (76.1%)	59 (18.6%)	17(5.6%)	0 (0%)	82 (79.6%)	15 (14.6%)	6 (5.8%)	0 (0%)	0.65
Muscle pain	276 (86.8%)	30 (9.4%)	12 (3.8%)	0 (0%)	94 (91.3%)	7 (6.8%)	2 (1.9%)	0 (0%)	0.454
Joint pain	295 (92.8%)	17 (5.3%)	6 (1.9%)	0 (0%)	100 (97.1%)	3 (2.9%)	0 (0%)	0 (0%)	0.216
Hypotension	311 (97.8%)	6 (1.9%)	1 (0.3%)	0 (0%)	97 (94.2%)	6 (5.8%)	0 (0%)	0 (0%)	0.097
Breathlessness	315 (99.1%)	2 (0.6%)	1 (0.3%)	0 (0%)	99 (96.1%)	4 (3.9%)	0 (0%)	0 (0%)	0.046
Nausea and vomiting	310 (97.5%)	4 (1.3%)	4 (1.3%)	0 (0%)	100 (97.1%)	2 (1.9%)	1 (1%)	0 (0%)	0.856
Diarrhea	316 (99.4%)	1 (0.3%)	1 (0.3%)	0 (0%)	100 (97.1%)	3 (2.9%)	0 (0%)	0 (0%)	0.53
Injection site pain	199 (62.6%)	78 (24.5%)	41 (12.9%)	0 (0%)	71 (68.9%)	20 (19.4%)	12 (11.7%)	0 (0%)	0.482
Injection site swelling	284 (89.3%)	28 (8.8%)	6 (1.9%)	0 (0%)	93 (90.3%)	8 (7.8%)	2 (1.9%)	0 (0%)	0.948
Allergic reaction	312 (98.1%)	3 (0.9%)	2 (0.6%)	1 (0.3%)	100 (97.1%)	2 (1.9%)	1 (1%)	0 (0%)	0.774
Tachycardia	315 (99.1%)	3 (0.9%)	0 (0%)	0 (0%)	99 (96.1%)	3 (2.9%)	1 (1%)	0 (0%)	0.072
Menstrual cycle disturbances	310 (97.5%)	7 (2.2%)	0 (0%)	1 (0.3%)	99 (96.1%)	4 (3.9%)	0 (0%)	0 (0%)	0.554
Headache	283 (89%)	17 (5.3%)	17 (5.3%)	1 (0.3%)	91(88.3%)	6 (5.8%)	6 (5.8%)	0 (0%)	0.942

Furthermore, the incidence of self-reported side effects was compared between the types of COVID-19 vaccines, and the results have been depicted in Table 8. When the mRNA vaccine was compared to other vaccines (inactivated and viral vector), mRNA vaccine recipients reported a significantly greater incidence of self-reported fever, headache, injection site pain, and injection site swelling. mRNA vaccines were also associated with a nonsignificant greater risk of hypotension, fatigue, muscle pain, joint pain, breathlessness, nausea and vomiting, diarrhea, and allergic reactions.

**Table 8:** Incidence of self-reported side effects among participants who received mRNA, inactivated, and viral vector-based COVID-19 vaccine

Side effects	Incidence of side effects: risk ratio (95% CI)	Incidence of side effects: risk ratio (95% CI)	Incidence of side effects: risk ratio (95% CI)
	mRNA	Inactivated	Viral vector
Fever	1.621 (1.423-2.114)	0.612 (0.430-0.869)	0.890 (0.662-1.197)
Cough	0.735 (0.193-2.803)	2.341 (0.690-7.946)	0.502 (0.108-2.332)
Headache	1.950 (1.139-3.339)	0.895 (0.489-1.638)	0.475 (0.237-0.955)
Flu-like illness	0.801 (0.334-1.921)	1.621 (0.711-3.695)	0.753 (0.301-1.881)
Chills	0.953 (0.325-2.794)	0.937 (0.299-2.930)	1.115 (0.381-3.265)
Hypotension	1.471 (0.503-4.298)	1.041 (0.326-3.317)	0.602 (0.168-2.153)
Fatigue	1.367 (0.965-1.936)	0.813 (0.543-1.218)	0.856 (0.583-1.257)
Muscle pain	1.525 (0.913-2.548)	0.720 (0.390-1.329)	0.836 (0.474-1.475)
Joint pain	1.716 (0.816-3.607)	0.426 (0.150-1.210)	1.063 (0.486-2.323)
Breathlessness	2.288 (0.519-10.05)	1.756 (0.399-7.732)	-
Nausea and vomiting	2.095 (0.639-6.636)	0.878 (0.237-3.255)	0.446 (0.98-2.037)
Diarrhea	2.574 (0.435-15.237	1.561 (0.264-9.228)	-
Injection site pain	1.986 (1.545-2.553)	0.706 (0.515-0.968)	0.606 (0.440-0.833)
Injection site swelling	2.479 (1.406-4.37)	0.780 (0.408-1.494)	0.380 (0.174-0.830)
Allergic reactions	2.145 (0.585-7.870)	1.171 (0.297-4.607)	0.251 (0.032-1.986)
Tachycardia	0.686 (0.135-3.496)	3.127 (0.709-13.746)	0.335 (0.041-2.752)
Menstrual cycle disturbances	0.858 (0.263-2.803)	3.278 (1.060-10.132)	0.182 (0.024-1.399)

Inactivated vaccine recipients reported a statistically significant and considerably lower incidence of self-reported fever, and injection site pain when compared to other types of vaccines (mRNA and viral vector). On the contrary, a higher significant risk of menstrual cycle disturbances was observed. Inactivated vaccines were associated with a greater incidence of cough, flu-like illness, hypotension, breathlessness, diarrhea, allergic reactions, and tachycardia, but these side effects were not significantly associated.

Moreover, viral vector vaccine recipients reported a nonsignificant lower incidence of the majority of side effects when compared to other vaccines (mRNA and inactivated), but headaches, injection site pain, and injection site swelling were significantly less reported. Viral vector vaccines were only associated with a greater risk of chills and joint pain, but no statistical significance was observed.

The severity of side effects produced by different types of COVID-19 vaccines was compared as shown in Table 9. Most of the side effects reported no significant association with the type of vaccines except fever, injection site pain, injection site swelling, and joint pain (p < 0.05). In terms of the severity of vaccine side effects (“No side effect,” “Mild,” “Moderate,” and “Severe”), mRNA vaccine produced greater severity compared to the other two. It was noted that the severity of injection site pain was associated with the type of vaccine, out of which mRNA vaccine produced greater severity (mild = 29%, moderate = 23.2%) followed by inactivated (mild = 20.6%, moderate = 7.1%), and viral vector (mild = 19.3%, moderate = 5.7%). The severity of fever was also observed to be significantly associated with the type of vaccine inoculated; the mRNA vaccine produced greater severity of this side effect out of all vaccines (mild = 14.8%, moderate = 28.4%) followed by the viral vector (mild = 14.3%, moderate = 16.4%) and inactivated vaccine (mild = 16.7%, moderate= 7.1%). Additionally, the association between joint pain and types of the vaccine was observed, where mRNA vaccine recipients reported greater severity (mild = 4.5%, moderate = 3.9%) when compared to viral vector (mild = 6.4%) and inactivated vaccine (mild = 3.2%), with later two reporting no moderate side effect. A significant association was also considerable between injection site swelling and types of vaccine, and greater severity of swelling was observed in mRNA vaccine recipients (mild = 13.5%, moderate = 3.2%), whereas inactivated vaccine recipients reported less severity (mild = 6.3%, moderate = 2.4%), and viral vector reported the least severity of the side effect (mild = 5%). Compared to the other vaccines, fatigue and headache were predominantly severely reported by mRNA vaccine recipients. However, this association was not statistically proven. Moreover, it was observed that viral vector vaccines produced the least severe side effects when compared to other vaccines, and some of the side effects like breathlessness and diarrhea were not reported by the recipients.

**Table 9:** Difference between the severity of self-reported side effects among the participants who received mRNA, inactivated, and viral vector-based COVID-19 vaccines

	Type of vaccine											
	Inactivated				mRNA				Viral vector			
Side effect	No side effect	Mild	Moderate	Severe	No side effect	Mild	Moderate	Severe	No side effect	Mild	Moderate	Severe
Fever	96 (76.2%)	21 (16.7%)	9 (7.1%)	0 (0%)	88 (56.8%)	23 (14.8%)	44 (28.4%)	0 (0%)	97 (69.3%)	20 (14.3%)	23 (16.4%)	0 (0%)
Chills	122 (96.8%)	4 (3.2%)	0 (0%)	0 (0%)	150 (96.8%)	3 (1.9%)	2 (1.3%)	0 (0%)	135 (96.4%)	4 (2.9%)	1 (0.7%)	0 (0%)
Cough	121 (96%)	5 (4%)	0 (0%)	0 (0%)	152 (98.1%)	2 (1.3%)	1 (0.6%)	0 (0%)	138 (98.6%)	1 (0.7%)	1 (0.7%)	0 (0%)
Headache	113 (89.7%)	9 (7.1%)	4 (3.2%)	0 (0%)	130 (83.9%)	9 (5.8%)	15 (9.7%)	1 (0.6%)	131 (93.6%)	5 (3.6%)	4 (2.9%)	0 (0%)
Flu-like illness	117 (92.9%)	6 (4.8%)	3 (2.4%)	0 (0%)	148 (95.5%)	3 (1.9%)	4 (2.6%)	0 (0%)	134 (95.7%)	4 (2.9%)	2 (1.4%)	0 (0%)
Hypotension	122 (96.8%)	4 (3.2%)	0 (0%)	0 (0%)	149 (96.1%)	5 (3.2%)	1 (0.6%)	0 (0%)	137 (97.9%)	3 (2.1%)	0 (0%)	0 (0%)
Fatigue	100 (79.4%)	18 (14.3%)	8 (6.3%)	0 (0%)	113 (72.9%)	30 (19.4%)	12 (7.7%)	0 (0%)	111 (79.3%)	26 (18.6%)	3 (2.1%)	0 (0%)
Muscle pain	114 (90.5%)	8 (6.3%)	4 (3.2%)	0 (0%)	131 (84.5%)	15 (9.7%)	9 (5.8%)	0 (0%)	125 (89.3%)	14 (10%)	1 (0.7%)	0 (0%)
Joint pain	122 (96.8%)	4 (3.2%)	0 (0%)	0 (0%)	142 (91.6%)	7 (4.5%)	6 (3.9%)	0 (0%)	131 (93.6%)	9 (6.4%)	0 (0%)	0 (0%)
Breathlessness	122 (96.8%)	4 (3.2%)	0 (0%)	0 (0%)	152 (98.1%)	2 (1.3%)	1 (0.6%)	0 (0%)	140 (100%)	0 (0%)	0 (0%)	0 (0%)
Nausea and vomiting	123 (97.6%)	2 (1.6%)	1 (0.8%)	0 (0%)	149 (96.1%)	2 (1.3%)	4 (2.6%)	0 (0%)	138 (98.6%)	2 (1.4%)	0 (0%)	0 (0%)
Diarrhea	124 (98.4%)	2 (1.6%)	0 (0%)	0 (0%)	152 (98.1%)	2 (1.3%)	1 (0.6%)	0 (0%)	140 (100%)	0 (0%)	0 (0%)	0 (0%)
Injection site pain	91 (72.2%)	26 (20.6%)	9 (7.1%)	0 (0%)	74 (47.7%)	45 (29%)	36 (23.2%)	0 (0%)	105 (75%)	27 (19.3%)	8 (5.7%)	0 (0%)
Injection site swelling	115 (91.3%)	8 (6.3%)	3 (2.4%)	0 (0%)	129 (83.2%)	21 (13.5%)	5 (3.2%)	0 (0%)	133 (95%)	7 (5%)	0 (0%)	0 (0%)
Allergic reactions	123 (97.6%)	2 (1.6%)	1 (0.8%)	0 (0%)	150 (96.8%)	2 (1.3%)	2 (1.3%)	1 (0.6%)	139 (99.3%)	1 (0.7%)	0 (0%)	0 (0%)
Tachycardia	122 (96.8%)	3 (2.4%)	1 (0.8%)	0 (0%)	153 (98.7%)	2 (1.3%)	0 (0%)	0 (0%)	139 (99.3%)	1 (0.7%)	0 (0%)	0 (0%)
Menstrual cycle disturbances	119 (94.4%)	6 (4.8%)	0 (0%)	1 (0.8%)	151 (97.4%)	4 (2.6%)	0 (0%)	0 (0%)	139 (99.3%)	1 (0.7%)	0 (0%)	0 (0%)

However, most of the side effects like allergic reactions, tachycardia, breathlessness, diarrhea, etc. were barely reported in terms of their severity for all the vaccines. The side effects that required hospital care (“severe”) were reported only by 0.7% of all the participants, with recipients of mRNA vaccine reporting 0.4%, inactivated vaccine reporting 0.2%, and viral vector reporting no severe side effect.

## Discussion

This study compared the reactogenicity of all types of COVID-19 vaccines available in Pakistan and their associated risk factors, which makes it the first ever of its kind to be conducted in the country. The majority of the recipients did not report any vaccine-related side effects. Self-reported side effects of all types of vaccines were largely mild in nature and did not require any treatment. Only three participants reported one severe side effect requiring hospital care. The results of this study were contrary to the results of the phase 3 safety trials including the major trial conducted for the Pfizer-BioNTech COVID-19 vaccine as those trials reported greater and more severe side effects compared to this study conducted in Pakistan [15].

In this study, the female gender was associated with a greater incidence of self-reported side effects, and in multiple studies, it has been reported that COVID-19 vaccine female recipients were at a greater risk than males, to develop dose-related side effects [16,17]. Vaccines related to a majority of the diseases in this world including COVID-19 vaccines are more reactogenic for females compared to the other gender because females have a more robust immune response compared to males [18]. It was also found that younger participants (<33 years) had greater chances of developing adverse effects compared to older ones. This finding might be explained by the fact that younger people produce more type 1 interferon than older people. Interferon alpha is an immune response mediator, which plays a key role in the vaccines' response [19]. This finding is in accordance with most of the studies conducted all around the world, highlighting that almost all the types of COVID-19 vaccines caused more frequent side effects in younger individuals [20-22].

In our study, it was analyzed that largely recipients with a history of COVID-19 infection were not more prone to vaccine-related side effects in comparison with the subjects without prior infection. History of COVID-19 infection was not a significant risk factor for the majority of adverse reactions except hypotension. In our study, the majority of subjects with a previous history of COVID-19 infection were the recipients of inactivated-based vaccines (68%), and it also revealed that inactivated vaccines were associated with lower incidence and lesser severity of most of the vaccine-related side effects compared to other vaccines. This observation might explain the finding of our study that previous history of infection was not associated with a greater number of self-reported side effects. A similar outcome was observed in a cross-sectional analytical study conducted in Pakistan that only included subjects who were inoculated with the Sinopharm (Inactivated) vaccine, which reported that a history of COVID-19 infection was significantly associated with decreased incidence of post-vaccination side effects [23].

Moreover, it was observed that prior history of infection was not linked to greater severity of all the self-reported side effects produced after the vaccination except breathlessness (p < 0.05). Although there is no clear explanation, participants with previous infections might have been suffering from long COVID-19 or persistent breathlessness after acute infection, which might have worsened after being vaccinated [24,25]. None of the participants with prior history of COVID-19 infection developed any severe side effects or required hospital care. These observations were in contrast with the extensive vaccine recipient survey conducted in the United Kingdom, which stated that prior COVID-19 infection was associated with significantly higher incidence and severity of self-reported side effects [26].

In the current study, mRNA vaccines were associated with the greatest risk of developing post-vaccination side effects in comparison to the other two types. Pfizer and Moderna vaccines produced side effects with greater severity than any other vaccine in our study. Contrarily, some literature highlights that mRNA vaccines have a lesser chance of producing side effects than viral vector-based vaccines [21]. Interestingly, multiple types of research have depicted that mRNA vaccines are more reactogenic than inactivated vaccines [21,27]. Most of the side effects reported by recipients of these vaccines required medication or treatment from a medical professional. In our study, the majority of the mRNA vaccine recipients were of young age, which itself is a risk factor for the incidence of side effects as observed in a trial reported in the Lancet [28].

Inactivated vaccines caused more and more severe vaccine-related side effects than viral vector vaccines.This observation contradicts all the previous literature on the comparison of the COVID-19 vaccine's reactogenicity, but it might be explained by the gender bias in viral vector vaccine recipients. It was also found that recipients of inactivated vaccines had milder side effects and decreased incidence compared to recipients of mRNA-based vaccines. This outcome is in line with the previous studies, and those studies clearly explain that Sinopharm and SinoVac vaccines are associated with less incidence and severity of self-reported side effects compared to other vaccines [21,27]. When compared to the other two types of vaccines, inactivated vaccines were related to the greatest incidence of menstrual cycle disturbances. This side effect was not included in the majority of the vaccine’s clinical trials, so only less literature is available on this observation. A study conducted in the United States found that recipients suffered from a change in cycle length but no disturbances in menstruation [17].

It was found that viral vector vaccines were associated with the least incidence and severity of vaccine-related side effects compared to the other two types of vaccines. This observation is contrary to the study conducted in Saudi Arabia, which highlighted that viral vector vaccines were associated with a greater incidence of self-reported side effects [29]. This finding might be explained by the fact that in our study, 74.3% of the viral vector vaccine recipients were males. Previous literature has proved that the female gender is associated with higher incidence and increased severity of self-reported side effects compared to males [30]. Earlier on, it was reported that one of the viral vector vaccines, AstraZeneca Oxford, was associated with blood clot formation in some female recipients, and these reports discouraged the use of that vaccine in females, especially young ones across the globe including Pakistan [20]. As a result, in our study, fewer responses from female recipients of viral vector vaccines, especially AstraZeneca, were reported.

Our study has various strengths, the most important of which is that it is the first designed report to compare and evaluate the severity of side effects produced by all COVID-19 vaccines available in Pakistan, despite the fact that a few studies have only evaluated the adverse effects of Sinopharm vaccine in Pakistani populations. This study is also one of the few independent studies that compared the safety profile of all the available COVID-19 vaccines worldwide. Another contribution of our study is that it sheds light on less commonly reported side effects like menstrual cycle disturbance, which is a global concern among female recipients but is rarely addressed in previous studies. This study also includes information about the association between certain medical risk factors and the severity of post-vaccination side effects. Furthermore, our study included participants who had a history of COVID-19 infection and suffered from various stages of infection (“asymptomatic,” “mild,” “moderate,” “severe,” and “critical”). This can be helpful to rule out vaccine hesitancy among people who had either stage of prior COVID-19 infection.

One of the methodological limitations is an unequal representation of males and females in this study. Moreover, the small number of subjects with prior history of COVID-19 infection suggests a study with equal representation of previously infected and non-infected participants for an adequate comparison of the severity of COVID-19 vaccine-related side effects. No specific data regarding the timing of the onset of side effects with the dose (whether first, second, or both) of the vaccine was obtained. In our study, few of the participants had received two doses of AstraZeneca (viral vector vaccine).

Nearly three fourth of individuals who received viral vector vaccines were males and the majority of mRNA recipients were of younger age, which might have impacted our study findings. So, further research should be conducted in Pakistan to compare the vaccine's reactogenicity among an equal proportion of individuals from both genders and similar age groups. In light of the results presented above, the government should run vaccine awareness campaigns highlighting that all types of vaccines used in Pakistan produce mild to moderate side effects, especially for individuals who have been previously infected, so that country can avoid the upcoming waves of this pandemic. Government should also use these findings to advertise all different vaccines being used in the vaccination drives to reduce hesitancy toward certain types of COVID-19 vaccines among the Pakistani population. Community-based non-governmental organizations (NGOs) should use this research to advocate and increase awareness for COVID-19 and various other vaccines in different communities present in all of the provinces included in our study.

## Conclusions

Our study reports that previous history of COVID-19 infection was not significantly associated with greater severity and increased risk of experiencing the majority of the vaccine-related side effects. This finding can be helpful to reduce hesitancy toward vaccines among people who have previously been infected with COVID-19. When comparing different vaccines, our data shows that mRNA vaccines produced greater severity and incidence of side effects, followed by inactivated and then viral vector-based vaccines. This is inconsistent with other studies, which report greater severity and incidence of side effects produced by viral vector vaccines. A small percentage of participants (0.7%) reported only one severe side effect, and the majority of the side effects reported in the study were mild, with few being moderate. Furthermore, young age and female gender were significantly associated with post-vaccination side effects. More studies analyzing the reactogenicity of all types of COVID-19 vaccines are necessary to acknowledge vaccine safety and boost public confidence in vaccination during this pandemic.
